# Hematogenous Macrophages: A New Therapeutic Target for Spinal Cord Injury

**DOI:** 10.3389/fcell.2021.767888

**Published:** 2021-11-24

**Authors:** Yuanzhe Ding, Di Zhang, Sheng Wang, Xiaolei Zhang, Jingquan Yang

**Affiliations:** ^1^ Department of Orthopaedics, The Second Affiliated Hospital and Yuying Children’s Hospital of Wenzhou Medical University, Wenzhou, China; ^2^ The Second School of Medicine, Wenzhou Medical University, Wenzhou, China; ^3^ Zhejiang Provincial Key Laboratory of Orthopedics, Wenzhou, China; ^4^ Chinese Orthopaedic Regenerative Medicine Society, Hangzhou, China

**Keywords:** spinal cord injury, hematogenous macrophages, microglia, inflammation, therapy

## Abstract

Spinal cord injury (SCI) is a devastating disease leading to loss of sensory and motor functions, whose pathological process includes mechanical primary injury and secondary injury. Macrophages play an important role in SCI pathology. According to its origin, it can be divided into resident microglia and peripheral monocyte-derived macrophages (hematogenous Mφ). And it can also be divided into M1-type macrophages and M2-type macrophages on the basis of its functional characteristics. Hematogenous macrophages may contribute to the SCI process through infiltrating, scar forming, phagocytizing debris, and inducing inflammatory response. Although some of the activities of hematogenous macrophages are shown to be beneficial, the role of hematogenous macrophages in SCI remains controversial. In this review, following a brief introduction of hematogenous macrophages, we mainly focus on the function and the controversial role of hematogenous macrophages in SCI, and we propose that hematogenous macrophages may be a new therapeutic target for SCI.

## Background

Spinal cord injury (SCI) used to be considered a traumatic disease that mostly occurs in the youth (Pickett et al., 2006; Niemeyer et al., 2020). However, the incidence of SCI among the elder people has gradually increased in recent years due to the aging of the population (Wilson et al., 2020). It is estimated that 270,000 people suffer from SCIs in the United States (DeVivo and Chen 2011; Selvarajah et al., 2014). SCI patients may experience two stages: the acute stage and the chronic stage (Oyinbo 2011). Recent therapies for SCI include pharmacological therapies, genetic therapies, cell therapies, and endocrine therapies (Samantaray et al., 2016; Przekora and Juszkiewicz 2020).

Notably, macrophages are related to all these therapies (Popovich 1999; Shechter 2009; Greenhalgh and David 2014; Chen et al., 2021; Zheng et al., 2021), which may be attributed to their different performances in the acute phase and chronic phase. When SCI happens, there are two groups of macrophages participating in the pathophysiological process. The first group is “microglia,” which is regarded as a tissue resident macrophage, and the other one is hematogenous macrophage, which is derived from monocytes circulating in the peripheral vessel (Hanisch and Kettenmann 2007; Daneman 2018; Li and Barres 2018). Hematogenous macrophages infiltrate from the periphery to the lesion site after SCI, through the damaged blood–spinal barrier and blood vessels (Hao et al., 2021). Their phenotypes will change dynamically, which may further modulate the inflammation, phagocytosis, scar formation, and regeneration of SCI (Sica 2015; Kong and Gao 2017). Current studies have shown that macrophages have advantages in that they can reduce spinal cord inflammation and phagocytize tissue debris (Lech and Anders 2013) in preparation for nerve regeneration and matrix remodeling. However, uncontrollable inflammatory response that they bring can cause secondary damage and can impair long-lasting recovery (Shechter et al., 2011; Zhu 2015).

In this review, we will summarize the recent advances about hematogenous macrophages, for the purpose of discovering a new therapeutic target for SCI.

## Hematogenous Macrophages Are Different From Microglia

### Hematogenous Macrophage’s Origin and Development

**FIGURE 1:**
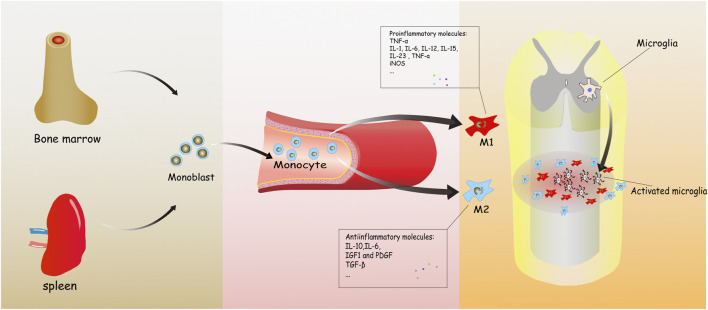
It concisely shows the development process of the monocyte-derived macrophages from bone marrow to injured spinal cord. Monoblasts, origin from the bone marrow, circulate in the blood normally. When inflammation happens, various molecules are released to induce and transform hematogenous macrophages into two types, playing mostly different roles in spinal cord injury (SCI).

Hematogenous macrophages are derived from monocytes. Bone marrow and the spleen are recently reported to be the two main origins of hematogenous macrophages (Ren and Young 2013; Swirski et al., 2014). Bone marrow-derived monocytes are defined as three subtypes in humans, which includes classical CD14^++^CD16^−^CCR2^+^, intermediate CD14^++^CD16^+^CCR2^+^, and nonclassical CD14^+^CD16^++^CCR2^−^ monocytes (Urbanski et al., 2017; Shang et al., 2021). The transformation of monocytes into macrophages and migration to tissues depends on inflammatory circumstance (Figure 1).

The spleen is now regarded as another major resource of monocytes. Blomster indicated that hematogenous macrophages mainly originate from splenic monocytes at the first 7 days post-injury (Blomster et al., 2013). Though splenic monocytes cannot be distinguished from counterparts that circulate in the blood vessels (Daneman 2018), further experiments found that they were spleen-resident monocytes rather than passing through the spleen within blood (Swirski and Nahrendorf 2009). Splenic monocytes can be divided into two subtypes including pro-inflammatory Ly-6C^+^ and anti-inflammatory Ly-6C^−^. After splenectomy, there is a reduction by about 75% of Ly-6C^+^ monocytes at the margin of myocardium infarction, which gave evidence that splenic macrophages play a critical role in inflammatory sites (Swirski and Nahrendorf 2009). What is more, according to the different anatomical parts of spleen, there are three different subsets of macrophages, namely, red pulp macrophage (RpMφ), marginal metallophilic macrophage (MMMφ), and marginal zone macrophage (MZMφ) (Mebius and Kraal 2005). They recognize aging or apoptotic red blood cells and eliminate them through SIRPα binding with CD47, as well as participate in pathogen-induced immune response (Burger et al., 2012). However, the relationship between spleen and SCI is unclear and still need further researches.

In physiological conditions, hematogenous macrophages make less contribution to tissue-resident macrophages than in pathological conditions (Hashimoto and Chow 2013). Hematogenous macrophages may infiltrate in different tissues in various situations, and this process is regulated by different secreted bioactive substances and enzymes (Margeta et al., 2018). For instance, in the breast tumor region lacking blood vessels, tumor cells may release cytokines such as vascular endothelial growth factor (VEGF), IL-8, and TGF-β to attract macrophages due to hypoxia, thus enabling them to play a role in promoting angiogenesis in tumor tissue (Kuroda and Jamiyan 2021). In obesity condition, insulin resistance may induce the increased expression of monocyte chemotactic protein 1 (MCP1), thereby promoting macrophage infiltration and inflammation in adipose tissue (Shimobayashi et al., 2018). In addition, during tissue hypoxia, the expression of activating transcription factor 4 (ATF4) increased, promoting the infiltration of M2 macrophages (Xia et al., 2017). In the SCI, pro-inflammatory cytokines such as IL-β, TNF-α, and IL-6, which are released by activated microglia cells (Jeong 2010), may promote the infiltration of hematogenous macrophages to the injury site.

### Microglia’s Origin and Development

“Microglia” is different from “hematogenous macrophages,” regarded as tissue-resident macrophages to participate in homeostasis in the central nervous system (CNS) (Zhao et al., 2018). Microglia and hematogenous macrophages have similar morphology and are both involved in serial pathological processes of SCI such as inflammation phagocytosis and ischemic reperfusion injury (Nakajima et al., 2020). However, they also have many differences in origin, biological markers, and functions (Daneman 2018; Watanabe et al., 2019).

Distinct from hematogenous macrophages, microglia originates from the yolk sac of the embryo (Ginhoux and Guilliams 2016) and apparently is generated earlier than hematogenous macrophages (Gomez Perdiguero et al., 2013). Microglia is found mainly in immunologically privileged sites such as the brain, spinal cord, and eyeball (Morganti-Kossmann et al., 2007). Microglia dynamically detects surrounding microenvironment without interfering with neuronal activities (Prinz et al., 2021). It can be the first to be activated when small blood vessels and parenchyma are damaged due to acute or chronic injury (Denes et al., 2007), followed by infiltration of hematogenous macrophages (Feng et al., 2018). Meanwhile, some studies have shown that the distribution of these two types of macrophages after infiltration is different. Microglia cells are distributed in the epicenter and edge of the lesion, while most hematogenous macrophages remained at the edge of the injury center (Shechter 2009) (Table 1).

**TABLE 1:** Distinction between hematogenous macrophage and microglia.

	Hematogenous macrophage	Microglia
Origin (Milich et al., 2019)	Myeloid progenitor cells	Yolk sac of the embryo
Location	Peripheral blood and tissue and margin of the lesion site after SCI (Bellver-Landete 2019)	Central nervous system and lesion core after SCI (Kroner and Rosas Almanza 2019)
Morphology (Milich, Ryan and Lee 2019)	Lager	Small volume
marker	CD11b^+^/CD45^+^/CX3CR1^−^/CCR2^+^/CD163 (Lu et al., 2018; Glavind et al., 2020)/P2Y1 (Chekeni et al., 2010)	CD11b^−^/CD45^−/^TMEM119/CX3CR1^+/^P2Y12/HIF-1α (Lin et al., 2020; Fu et al., 2021)
Phagocytosis capacity (Green et al., 2016)	More efficient phagocytosis	Transient phagocytosis

Note. SCI, spinal cord injury.

## Hematogenous Macrophages Participate in Spinal Cord Injury Pathology

### Inflammation

Inflammation is the most important pathological process after SCI with complex mechanism (Perdiguero et al., 2011). Damaged tissue fragments, nucleotide-binding oligomerization domain (NOD) (Inohara and Nunez 2003), and heat shock proteins (HSPs) (Khandia et al., 2017) act as inflammatory stimulus, which work on Toll-like receptors (TLRs) (Gao et al., 2021) and mannose receptors (Zhou et al., 2018). Under the synergistic effect of the above factors, hematogenous macrophages are recruited from the peripheral through the damaged blood–spinal barrier (Gensel and Zhang 2015).

So far, two phenotypes of hematogenous macrophages have been described: M1 and M2. M1 macrophages are firstly polarized under the stimulation of TNF-α, IL-β, and lipopolysaccharide (LPS) in the early phase of inflammation (Sica 2015). Meanwhile, they release inflammatory cytokines like IL-β,TNF-α, IL-6 (Jeong 2010), chemokines (CCL8, CCL9, and CCL15) (Boche et al., 2013), inducible nitric oxide synthase (iNOS), and cox-oxidase (COX) (Han et al., 2019). All the above can be regarded as characteristics of M1 macrophages. Meanwhile, M1 macrophages showed stronger phagocytosis and antigen-presenting ability, which could eliminate the necrotic cells (Hou et al., 2020). However, excessive secretion of pro-inflammatory cytokines, reactive oxygen species (ROS), and reactive nitrogen species (RNS) after M1 cell polarization can impair neurons and glia and even cause more serious neuron apoptosis (Block et al., 2007).

M2 macrophages are stimulated mainly by IL-4, IL-10, and TGF-β (Mills 2012), which can inhibit the apoptosis of neurons and inflammatory reaction, which consequently promotes the repair of nerve tissue (Zhang et al., 2020). They have lower expression of the pro-inflammatory factors as compared with M1 macrophages (Motwani and Gilroy 2015). M2 macrophages can be divided into four subtypes according to different cell definition markers: M2a, M2b, M2c, and M2d. M2a macrophages can be marked by CD206, CD209, arginase-1, and YM1, which contribute to remyelination and reduction of dieback (Novak and Koh 2013). M2b macrophages are also called regulatory macrophages (Mregs), expressing CD86 and CD64. Different from other subtypes, they have both anti-inflammatory and pro-inflammatory functions (Nakai 2021). M2c macrophages can express CD163, CD206, and CCR2. They function as debris scavenging and remyelination (Novak and Koh 2013). M2d is typically regarded as tumor-associated macrophage (TAM), expressing vascular endothelial growth factor (VEGF) and promoting angiogenesis and tumor growth (Kuroda and Jamiyan 2021).

### Phagocytosis

Hematogenous macrophages detect, engulf, and digest cellular and tissue debris in the process of phagocytosis (Jain et al., 2019). Fragments persist longer in hematogenous macrophages, which shows hematogenous macrophages have less efficient phagocytic capability and are more susceptible than microglia (Greenhalgh and David 2014). Myelin debris are inflammatory stimuli and neural outgrowth inhibitors generated after SCI; they also contain high amounts of lipid (Dimas et al., 2019). Foamy macrophage is another phenotype of hematogenous macrophage after phagocytizing myelin lipid. Studies recently have demonstrated that foamy macrophage may lose the capacity to phagocytize apoptotic cells and promote the release of pro-inflammatory cytokines (Wang 2015). Kong et al. indicated that macrophage scavenger receptor 1 (MSR1) participated in the formation of foamy macrophages accompanied with activating the NF-κB signaling pathway (Kong et al., 2020).

Furthermore, studies have shown that phagocytosis may have different effects in SCI. On the positive side, phagocytosis can provide a suitable environment for remyelination via receptor-mediated phagocytosis (McKerracher and Rosen 2015), because myelin debris produce regeneration inhibitors and inflammatory stimulus (He and Koprivica 2004). On the negative side, phagocytosis may lead to axonal dieback. Cx3cr1+/GFP hematogenous macrophages are involved in the process by directly contacting injured axons (Evans et al., 2014). What is more, Popovich *et al.* indicated that depleting hematogenous macrophages by clodronate liposomes decreased axonal dieback (Popovich 1999).

### Glial Scar Formation

Astrocyte-producing chondroitin sulfate proteoglycans (CSPGs) contribute to the glial scar formation after SCI (Haan et al., 2015; Vismara et al., 2020). Researchers believe that an interaction exists between macrophages and CSPGs, because there are similar temporal and spatial characteristics between them (Song et al., 2019). Compared with normal mice, scar tissue in genetically engineered mice without macrophages was significantly reduced (Martin et al., 2003). In addition, scar formation is related to the macrophage subtypes (Hesketh et al., 2017). M1 macrophages show anti-fibrotic behavior but promote inflammation (Sun et al., 2020), while M2 macrophages induce scar formation on account of TGF-β secretion (Song 2019). Scar tissue has shown different effects after SCI. In the acute phase, scar tissue acts as a barrier to restrict the inflammation. This process can be dependent on STAT3 signaling pathway within astrocytes, while STAT3-deficient mice failed to corral inflammation (Wanner et al., 2013). Sahni *et al.* have indicated that bone morphogenetic protein receptor Ia (BMPRIa) contributes to gliosis and that BMPRIa ablation leads to less axon density and worse locomotor recovery after SCI (Sahni et al., 2010). In the chronic phase, the scar starts to show detrimental effects as a barrier for axonal regrowth (Dias 2018). Undesirable regenerative conditions can be attributed to the hostile scar tissue rather than the limited regeneration capacity of axons (Tom et al., 2004). Leukocyte common antigen-related phosphatase (LAR) is highly correlated with CSPGs in scar tissues. Xu et al. have indicated that LAR knockout mice show improvements in not only axonal regeneration but also functional recovery (Xu et al., 2015). What is more, type A pericytes, a subset of perivascular cells, have now come into view regarding their contribution to extracellular matrix deposition and scar composition (Picoli et al., 2019). Dias *et al.* have indicated that moderate inhibition of pericyte-derived scar formation can facilitate wound healing integrity as well as axonal regeneration (Dias 2018).

### Regeneration

As mentioned above, macrophage-induced inflammation, phagocytosis, and scar formation are all like double-edged swords to SCI recovery. Likewise, regeneration can also be attributed to macrophage phenotypes (Wu et al., 2015; Zrzavy et al., 2021). From the perspective of macrophage phenotypes, it has been clear that macrophages can be activated into M1 and M2, and simply reducing macrophages without differentiating phenotypes at the damaged site is not conducive to the regeneration (Shechter 2009; Miron et al., 2013). When circulating pro-inflammatory M1 macrophages are eliminated, inflammation is attenuated and neuroprotective effects are shown (Jay 2015; Wang et al., 2019). However, Ma and colleagues demonstrated that M2 macrophage transplantation contributed to a better preservation of myelinated axons. What is more, M2 expresses fibroblast growth factor (Fgf2) and insulin-like growth factor-1 (Igf1) to stimulate angiogenesis, which is a crucial element to provide an environment promoting nerve regeneration (Jetten et al., 2014; Hu et al., 2019). Likewise, the p38/MAKP-1 pathway is involved in the transition of macrophages from an “inflammatory” to “anti-inflammatory” role, impairing inflammation and ameliorating the tissue repair (Song et al., 2021).

## Controversy Over the Treatment of Spinal Cord Injury by Hematogenous Macrophages

Due to the contradictory views on the influences of hematogenous macrophages in SCI, researchers are divided into two groups. They hold different views that infiltrating hematogenous macrophages after SCI have beneficial and detrimental effects. These views are listed as follows.

### Hematogenous Macrophages May Inhibit Spinal Cord Injury Repair

Numerous studies demonstrated that reducing infiltration of hematogenous macrophages or clearing them at the injured site promotes the recovery of SCI.

Based on the previous evidence that macrophages are related to secondary damage, Blight conducted an experiment by injecting silica dust into animals at 2 days after SCI, which shows less vascularization of the lesion (Blight 1985). What is more, Popovich intravenously injected clodronate liposome to deplete hematogenous macrophages, which led to a reduction of infiltrating macrophages at the damaged edge. As a result, it decreased the tissue cavity in lesion and promoted the recovery of motor function (Popovich 1999). Arising from the hypothesis that macrophages may participate in scar formation, Zhu and colleagues applied the same approach with Popovich, getting the result that the density of neurofilament axon increased as compared with control group (Zhu 2015).

Instead of depleting hematogenous macrophages, Gris and colleagues chose the CD11d monoclonal antibody (mAb) to delay macrophage’s infiltration and to interfere with the early inflammatory response; the results showed that necrotic debris are significantly reduced and long-lasting sensorimotor function is improved. Compared with Popovich’s experiment, this method does not affect later aggregation of macrophages crucial for regeneration (Parvin et al., 2021). Besides the methods above, Mabon used an antibody to block the binding of αDβ2–VCAM1 in order to reduce recruitment of macrophages and neutrophils (Mabon 2000). Adiponectin, a hormone secreted by adipocytes, is also able to inhibit macrophage recruitment as well as its mediated neuroinflammation (Zhou 2019). Likewise, MCP-1 (Yang et al., 2018), TNF-α, and macrophage inflammatory protein 1 (MIP) (Maurer and von Stebut 2004) may also cause the infiltration of hematogenous macrophages. Inhibiting the infiltration of hematogenous macrophages shares the same results in that they controlled the inflammation and reduced myelin lipid accumulation, which can have potential for long-lasting sensorimotor function recovery in SCI (Huang 2019).

### Hematogenous Macrophages Promote Spinal Cord Injury Repair

However, different from the above, many studies have demonstrated that hematogenous macrophages alleviate SCI.

Kobayakawa indicated that recruiting hematogenous macrophages to the lesion epicenter by high concentration of complement C5a leads to a lower incidence of axonal dieback and improvement of recovery, because after epicenter-directed accumulation, there will be less scattering hematogenous macrophages in lesions, which used to widely come into contact with neuron and cause axonal dieback (Kobayakawa 2019). In addition, hematogenous macrophages can secrete exosomes containing IL-10. Exosomes not only promote the polarization of anti-inflammatory microglia but also have neuroprotective effects and induce autophagy by downregulating the PI3K/AKT/mTOR signaling pathway (Huang 2019). Since M2 macrophages can produce anti-inflammatory cytokines and promote angiogenesis, Chen et al. adopted the method of transferring M2 to the injured spinal cord, which promoted neural development of injured spinal cord and inhibited neuronal death by regulating nucleoli and ribosome biogenesis (Chen et al., 2019). Similarly, in retinal injury, the direct transfer of naive monocytes to the injured mice also promotes neuroprotection and the renewal of retinal progenitor cells (London et al., 2011).

The above researches show the advantages of the infiltration of hematogenous macrophages; other researchers also have the same view by blocking the infiltration of hematogenous macrophages. For instance, interaction of monocyte chemoattractant protein-1 (MCP-1) with CCR2 is involved in the initial recruitment of hematogenous macrophages to the lesion (Zhang et al., 2021). When CCR2 was blocked, there was less infiltration of hematogenous macrophages, which leads to more myelin loss and worse recovery (Daneman 2018). What is more, Wattananit found that depleting hematogenous macrophages in the 7 days post-injury attenuates expression of anti-inflammatory gene like Ym1, TGF-βand CD163, while pro-inflammatory effects induced by microglia were increased (Wattananit et al., 2016) (Table 2).

**TABLE 2:** Different treatments towards hematogenous macrophages.

Objective	Treatment	Experimental principle	Result
To prove Mφ is detrimental (Huang 2019)	Silica dust (Blight 1985)	Exert cytotoxic effects to Mφ	Less myelin axons and less vascularization in the lesion
	Clodronate (Popovich 1999)	Deplete peripheral Mφ	Decreased the tissue cavity and promoted motor function
	Anti-CD11d mAb (Gris 2004)	Block the interaction between endothelial cell and hematogenous Mφ	Increasing density of neurofilament axon
	Anti-αDβ2 mAb (Mabon 2000; Naeini et al., 2021)	Block the connection of αDβ2–VCAM-1	Less necrotic debris and long-lasting sensorimotor function recovery
	Adiponectin (Zhou 2019)	Suppress myelin lipid accumulation	Reduced myelin lipid accumulation and impaired neurogenesis
To prove Mφ is beneficial	C5a (Dander et al., 2021)	Induce epicenter-directed macrophage migration	Avoid neuron contact and reduce incidence of axonal dieback
	Transplantation (Han et al., 2021)	Transfer M2 to injured spinal cord	Promote reactive oxygen species production and regeneration
	Anti-CCR2 antibody (Daneman 2018)	Selectively deplete the CD115 + CD11b + Ly6C+ monocytes	Greater myelin loss

### Analysis of Causes of the Controversy

In this review, we discuss a number of experiments about hematogenous macrophages in SCI, which reflect the pathophysiological role of hematogenous macrophages likes a “double-edged sword.” In fact, it is because the polarization and executive functions of hematogenous macrophages are time-dependent and dynamically changing while infiltrating into the lesion location. At 3 days post-injury, hematogenous macrophages begin to infiltrate the injured site and then dominate the lesion core (Longbrake et al., 2007). At 7 days after SCI, the number of M2 macrophages peaked. However, the degree of infiltration gradually decreased till 14 dpi, which can be attributed to lipid accumulation of myelin fragments (Wang 2015). Beck et al. found that this decrease was not permanent and that the second wave of increase in hematogenous macrophages happened from 14 to 60 dpi. They also have a hypothesis that the hematogenous macrophage response of this phase plays a critical role in preventing further loss of function (Beck et al., 2010). However, the macrophages’ role after 60 dpi is unclear.

What is more, polarization of hematogenous macrophages occurs during this dynamic process. The researchers advocating the elimination of hematogenous macrophages are focusing on detrimental effects, which are mediated by M1 macrophages, such as inflammation (Gris 2004; Arafah et al., 2019). Others advocating the benefits of hematogenous macrophages have paid attention to the anti-inflammatory and regenerative effects of M2 macrophages (Kobayakawa 2019). The transformation of hematogenous macrophage subtypes is considered to be a key therapeutic target (Novak and Koh 2013). But the transformation will not take place spontaneously, as the injured lesion microenvironment is more suitable for the survival of M1 rather than M2 (David et al., 2015). Thus, scientists are now trying to promote M2 macrophage polarization to secure SCI; here are the studies that have been reported up to now.

For example, mTOR and peroxisome proliferator-activated receptor-γ (PPARγ) are involved in metabolic programs of anti-inflammatory macrophages, which can promote the polarization of M2 macrophages. Inhibition of mTOR leads to a decreasing expression of PPARγ, thereby inhibiting polarization of M2 macrophages (Kang et al., 2018). Signal transducer and activator of transcription 6 (STAT6) is proved to participate in the progress of the infiltration as well as the polarization of M2 macrophages, which is a potential therapy for SCI (Zhou et al., 2020). Thus, Yao et al. used an immune inhibitory receptor called programmed cell death 1 (PD-1) to induce the phosphorylation of STAT6, which brings positive effects like removing debris and facilitating tissue repair (Yao et al., 2014). Liu et al. first established that the inhibited expression of gene PTEN in macrophages resulted in increased M2 polarization. Inhibiting PTEN by bovine papillomavirus (bpV) shows satisfactory effects including promoting axonal outgrowth and improving tissue sparing *in vivo* after SCI (Liu et al., 2019). What is more, Grb1/2-associated binder (Gab) proteins are components in response to various extracellular stimuli, which are a determinant in M2 macrophage polarization. Deficiency of Gab1/2 attenuates macrophage sensitivity to IL-4 and leads to a depression in M2 polarization (Guo et al., 2017). Researchers also indicated that local injection of brain-derived neurotrophic factor (BDNF) can activate the polarization of M2 macrophages via IL-10 and IL-13, which attenuates inflammatory microenvironment. Meanwhile, BDNF has also shown its contribution to synaptic plasticity and axon regeneration in SCI by the high-affinity TrkB receptor (Ji et al., 2015). Nuclear factor-kappaB (NF-κB) is a major pro-inflammatory regulator of macrophages. Parthenolide is the principal active ingredient of herbs, which can inhibit NF-κB pathway to promote M1 transfer to M2. This treatment shows suppressed glial scar formation and inhibition of demyelination (Gaojian et al., 2020). Ma et al. indicated that implanting *in vitro*-polarized M2 macrophages directly to the lesion site will be a better method, which inhibits ROS production and promotes regeneration (Ma et al., 2015), because they think it is too late for transferred cells to reach the lesion site when the blood–brain barrier has been closed (Hu et al., 2012). Obviously, the above methods can increase the proportion of M2 macrophages to achieve a significant therapeutic effect. It is believed that in the future it will be a principle to promote M2 macrophage polarization in clinical treatment for SCI.

## Summary and Perspectives

To sum up, the infiltrating and polarizing hematogenous macrophages show different functions at different times and states. As mentioned above, targeting infiltrating macrophages to treat SCI will be a major trend in the future. Simply clearing or promoting macrophages is not beneficial. The key is how to properly regulate their phenotypes. However, the methods for regulating polarization of hematogenous macrophages are still limited. It is necessary to find more ways to properly regulate the hematogenous macrophages after SCI. By then, there will be more evidences on the roles of macrophages in the treatment of SCI.

## Abbreviations

ATF4, activating transcription factor 4; CCR, chemokine receptor; CD, cluster of differentiation; CNS, central nervous system; CSF, colony-stimulating factor; CSPGs, chondroitin sulfate proteoglycans; DAMPs, damage-associated molecular patterns; HSP, heat shock proteins; IL, interleukin; Mφ, Macrophage; MCP1, monocyte chemotactic protein 1; MCP, monocyte chemotactic protein 1; MMP, matrix metalloproteinase; NOD, nucleotide-binding oligomerization domain; PPARγ, peroxisome proliferator-activated receptor-γ; SIRPα, signal regulatory proteinα; SCI, spinal cord injury; TGF, transforming growth factor; TNF, tumor necrosis factor; VEGF, vascular endothelial growth factor; TLRs, Toll-like receptors.

## References

[B1] Arafah M. M. Al-Qattan M. M. Abdulmaged-Ahmed D. A. Al-Nafesah G. A. Jadu N. Y. Bagayawa R. S. (2019). The Use of Antifibrotic Recombinant nAG Protein in a Rat Liver Fibrosis Model. Biomed. Res. Int. 2019, 1–5. 10.1155/2019/9846919 PMC6582902 31275996

[B2] Beck K. D. Nguyen H. X. Galvan M. D. Salazar D. L. Woodruff T. M. Anderson A. J. (2010). Quantitative Analysis of Cellular Inflammation after Traumatic Spinal Cord Injury: Evidence for a Multiphasic Inflammatory Response in the Acute to Chronic Environment. Brain Feb 133, 433–447. 10.1093/brain/awp322 PMC2858013 20085927

[B3] Bellver-Landete V. Bretheau F. Mailhot B. Vallières N. Lessard M. Janelle M.-E. (2019). Microglia Are an Essential Component of the Neuroprotective Scar that Forms after Spinal Cord Injury. Nat. Commun. 10, 518. 10.1038/s41467-019-08446-0 30705270 PMC6355913

[B4] Blight A. R. (1985). Delayed Demyelination and Macrophage Invasion: a Candidate for Secondary Cell Damage in Spinal Cord Injury. Cent. Nervous Syst. Trauma 2, 299–315. 10.1089/cns.1985.2.299 3836014

[B5] Block M. L. Zecca L. Hong J.-S. (2007). Microglia-mediated Neurotoxicity: Uncovering the Molecular Mechanisms. Nat. Rev. Neurosci. 8, 57–69. 10.1038/nrn2038 17180163

[B6] Blomster L. V. Brennan F. H. Lao H. W. Harle D. W. Harvey A. R. Ruitenberg M. J. (2013). Mobilisation of the Splenic Monocyte Reservoir and Peripheral CX3CR1 Deficiency Adversely Affects Recovery from Spinal Cord Injury. Exp. Neurol. 247, 226–240. 10.1016/j.expneurol.2013.05.002 23664962

[B7] Boche D. Perry V. H. Nicoll J. A. R. (2013). Review: Activation Patterns of Microglia and Their Identification in the Human Brain. Neuropathol. Appl. Neurobiol. 39, 3–18. 10.1111/nan.12011 23252647

[B8] Burger P. Hilarius-Stokman P. de Korte D. van den Berg T. K. van Bruggen R. (2012). CD47 Functions as a Molecular Switch for Erythrocyte Phagocytosis. Blood 119, 5512–5521. 10.1182/blood-2011-10-386805 22427202

[B9] Chekeni F. B. Elliott M. R. Sandilos J. K. Walk S. F. Kinchen J. M. Lazarowski E. R. (2010). Pannexin 1 Channels Mediate 'find-Me' Signal Release and Membrane Permeability during Apoptosis. Nature 467, 863–867. 10.1038/nature09413 20944749 PMC3006164

[B10] Chen F. Hu M. Shen Y. Zhu W. Cao A. Ni B. (2021). Isorhamnetin Promotes Functional Recovery in Rats with Spinal Cord Injury by Abating Oxidative Stress and Modulating M2 Macrophages/microglia Polarization. Eur. J. Pharmacol. 895, 173878. 10.1016/j.ejphar.2021.173878 33453223

[B11] Chen J. Wu Y. Duan F.-X. Wang S.-N. Guo X.-Y. Ding S.-Q. (2019). Effect of M2 Macrophage Adoptive Transfer on Transcriptome Profile of Injured Spinal Cords in Rats. Exp. Biol. Med. (Maywood) 244, 880–892. 10.1177/1535370219854668 31159561 PMC6690136

[B12] Dander E. Fallati A. Gulić T. Pagni F. Gaspari S. Silvestri D. (2021). Monocyte-macrophage Polarization and Recruitment Pathways in the Tumour Microenvironment of B‐cell Acute Lymphoblastic Leukaemia. Br. J. Haematol. 193, 1157–1171. 10.1111/bjh.17330 33713428

[B13] David S. Greenhalgh A. D. Kroner A. (2015). Macrophage and Microglial Plasticity in the Injured Spinal Cord. Neuroscience 307, 311–318. 10.1016/j.neuroscience.2015.08.064 26342747

[B14] Denes A. Vidyasagar R. Feng J. Narvainen J. McColl B. W. Kauppinen R. A. (2007). Proliferating Resident Microglia after Focal Cerebral Ischaemia in Mice. J. Cereb. Blood Flow Metab. 27, 1941–1953. 10.1038/sj.jcbfm.9600495 17440490

[B15] DeVivo M. J. Chen Y. (2011). Trends in New Injuries, Prevalent Cases, and Aging with Spinal Cord Injury. Arch. Phys. Med. Rehabil. 92, 332–338. 10.1016/j.apmr.2010.08.031 21353817

[B16] Dias D. O. Kim H. Holl D. Werne Solnestam B. Lundeberg J. Carlén M. (2018). Reducing Pericyte-Derived Scarring Promotes Recovery after Spinal Cord Injury. Cell 173, 153–165. 10.1016/j.cell.2018.02.004 29502968 PMC5871719

[B17] Dimas P. Montani L. Pereira J. A. Moreno D. Trötzmüller M. Gerber J. (2019). CNS Myelination and Remyelination Depend on Fatty Acid Synthesis by Oligodendrocytes. Elife 8, e44702. 10.7554/eLife.44702 31063129 PMC6504237

[B18] Evans T. A. Barkauskas D. S. Myers J. T. Myers E. G. You J. Q. Ransohoff R. M. (2014). High-resolution Intravital Imaging Reveals that Blood-Derived Macrophages but Not Resident Microglia Facilitate Secondary Axonal Dieback in Traumatic Spinal Cord Injury. Exp. Neurol. 254, 109–120. 10.1016/j.expneurol.2014.01.013 24468477 PMC3954731

[B19] Feng Y. Lu Y. Liu D. Zhang W. Liu J. Tang H. (2018). Apigenin-7- O -β- D -(-6″- P -Coumaroyl)-Glucopyranoside Pretreatment Attenuates Myocardial Ischemia/reperfusion Injury via Activating AMPK Signaling. Life Sci. 203, 246–254. 10.1016/j.lfs.2018.04.048 29705352

[B20] Fu M. Zhu Y. Zhang J. Wu W. Sun Y. Zhang X. (2021). MicroRNA-221-3p Suppresses the Microglia Activation and Seizures by Inhibiting of HIF-1α in Valproic Acid-Resistant Epilepsy. Front. Pharmacol. 12, 714556. 10.3389/fphar.2021.714556 34497517 PMC8419275

[B21] Gao Y. H. Wang J. Y. Han Y. J. Liu J. L. (2021). Spinal Cord Toll like Receptor 4 and its Co-stimulatory Molecule Heat Shock Protein 90 May Parti-Cipate in Electroacupuncture Analgesia in Rats with Chronic Neuropathic Pain. Zhen Ci Yan Jiu 46, 735–741. 10.13702/j.1000-0607.201103 34558238

[B22] Gaojian T. Dingfei Q. Linwei L. Xiaowei W. Zheng Z. Wei L. (2020). Parthenolide Promotes the Repair of Spinal Cord Injury by Modulating M1/M2 Polarization via the NF-Κb and STAT 1/3 Signaling Pathway. Cell Death Discov. 6, 97. 10.1038/s41420-020-00333-8 PMC7538575 33083018

[B23] Gensel J. C. Zhang B. (2015). Macrophage Activation and its Role in Repair and Pathology after Spinal Cord Injury. Brain Res. 1619, 1–11. 10.1016/j.brainres.2014.12.045 25578260

[B24] Ginhoux F. Guilliams M. (2016). Tissue-Resident Macrophage Ontogeny and Homeostasis. Immunity 44, 439–449. 10.1016/j.immuni.2016.02.024 26982352

[B25] Glavind E. Gotthardt D. N. Pfeiffenberger J. Sandahl T. D. Bashlekova T. Willemoe G. L. (2020). The Macrophage Activation Marker Soluble CD163 Is Elevated and Associated with Liver Disease Phenotype in Patients with Wilson's Disease. Orphanet J. Rare Dis. 15, 173. 10.1186/s13023-020-01452-2 32615997 PMC7331244

[B26] Gomez Perdiguero E. Schulz C. Geissmann F. (2013). Development and Homeostasis of "resident" Myeloid Cells: the Case of the Microglia. Glia 61, 112–120. 10.1002/glia.22393 22847963

[B27] Green D. R. Oguin T. H. Martinez J. (2016). The Clearance of Dying Cells: Table for Two. Cell Death Differ 23, 915–926. 10.1038/cdd.2015.172 26990661 PMC4987729

[B28] Greenhalgh A. D. David S. (2014). Differences in the Phagocytic Response of Microglia and Peripheral Macrophages after Spinal Cord Injury and its Effects on Cell Death. J. Neurosci. 34, 6316–6322. 10.1523/JNEUROSCI.4912-13.2014 24790202 PMC6608120

[B29] Greenhalgh A. D. Zarruk J. G. Healy L. M. Baskar Jesudasan S. J. Jhelum P. Salmon C. K. (2018). Peripherally Derived Macrophages Modulate Microglial Function to Reduce Inflammation after CNS Injury. Plos Biol. 16, e2005264. 10.1371/journal.pbio.2005264 30332405 PMC6205650

[B30] Gris D. (2004). Transient Blockade of the CD11d/CD18 Integrin Reduces Secondary Damage after Spinal Cord Injury, Improving Sensory, Autonomic, and Motor Function. J. Neurosci. 24, 4043–4051. 10.1523/JNEUROSCI.5343-03.2004 15102919 PMC6729422

[B31] Guo X. Li T. Xu Y. Xu X. Zhu Z. Zhang Y. (2017). Increased Levels of Gab1 and Gab2 Adaptor Proteins Skew Interleukin-4 (IL-4) Signaling toward M2 Macrophage-Driven Pulmonary Fibrosis in Mice. J. Biol. Chem. 292, 14003–14015. 10.1074/jbc.M117.802066 28687632 PMC5572909

[B32] Haan N. Zhu B. Wang J. Wei X. Song B. (2015). Crosstalk between Macrophages and Astrocytes Affects Proliferation, Reactive Phenotype and Inflammatory Response, Suggesting a Role during Reactive Gliosis Following Spinal Cord Injury. J. Neuroinflammation 12, 109. 10.1186/s12974-015-0327-3 26025034 PMC4457974

[B33] Han G. H. Kim S. J. Ko W. K. Lee D. Han I. B. Sheen S. H. (2021). Transplantation of Tauroursodeoxycholic Acid-Inducing M2‐phenotype Macrophages Promotes an Anti‐neuroinflammatory Effect and Functional Recovery after Spinal Cord Injury in Rats. Cell Prolif 54, e13050. 10.1111/cpr.13050 33960559 PMC8168422

[B34] Han Y. Guo W. Ren T. Huang Y. Wang S. Liu K. (2019). Tumor-associated Macrophages Promote Lung Metastasis and Induce Epithelial-Mesenchymal Transition in Osteosarcoma by Activating the COX-2/STAT3 axis. Cancer Lett. 440-441, 116–125. 10.1016/j.canlet.2018.10.011 30343113

[B35] Hanisch U.-K. Kettenmann H. (2007). Microglia: Active Sensor and Versatile Effector Cells in the normal and Pathologic Brain. Nat. Neurosci. 10, 1387–1394. 10.1038/nn1997 17965659

[B36] Hao D. Du J. Yan L. He B. Qi X. Yu S. (2021). Trends of Epidemiological Characteristics of Traumatic Spinal Cord Injury in China, 2009-2018. Eur. Spine J. 30, 3115–3127. 10.1007/s00586-021-06957-3 34392419

[B37] Hashimoto D. Chow A. Noizat C. Teo P. Beasley M. B. Leboeuf M. (2013). Tissue-resident Macrophages Self-Maintain Locally throughout Adult Life with Minimal Contribution from Circulating Monocytes. Immunity 38, 792–804. 10.1016/j.immuni.2013.04.004 23601688 PMC3853406

[B38] He Z. Koprivica V. (2004). The Nogo Signaling Pathway for Regeneration Block. Annu. Rev. Neurosci. 27, 341–368. 10.1146/annurev.neuro.27.070203.144340 15217336

[B39] Hesketh M. Sahin K. B. West Z. E. Murray R. Z. (2017). Macrophage Phenotypes Regulate Scar Formation and Chronic Wound Healing. Int. J. Mol. Sci. 18, 1545. 10.3390/ijms18071545 PMC5536033 28714933

[B40] Hou C. Mei Q. Song X. Bao Q. Li X. Wang D. (2020). Mono-macrophage-Derived MANF Protects against Lipopolysaccharide-Induced Acute Kidney Injury via Inhibiting Inflammation and Renal M1 Macrophages. Inflammation 44, 693–703. 10.1007/s10753-020-01368-w 33145627

[B41] Hu C. Bai X. Liu C. Hu Z. (2019). Long Noncoding RNA XIST Participates Hypoxia-Induced Angiogenesis in Human Brain Microvascular Endothelial Cells through Regulating miR-485/SOX7 axis. Am. J. Transl Res. 11, 6487–6497. Available from: https://www.ncbi.nlm.nih.gov/pubmed/31737200 31737200 PMC6834526

[B42] Hu J.-G. Shen L. Wang R. Wang Q.-Y. Zhang C. Xi J. (2012). Effects of Olig2-Overexpressing Neural Stem Cells and Myelin Basic Protein-Activated T Cells on Recovery from Spinal Cord Injury. Neurotherapeutics 9, 422–445. 10.1007/s13311-011-0090-9 22173726 PMC3337015

[B43] Huang H. Lai S. Luo Y. Wan Q. Wu Q. Wan L. (2019). Nutritional Preconditioning of Apigenin Alleviates Myocardial Ischemia/Reperfusion Injury via the Mitochondrial Pathway Mediated by Notch1/Hes1. Oxidative Med. Cell Longevity 2019, 1–15. 10.1155/2019/7973098 PMC6446095 31015891

[B44] Inohara N. Nuñez G. (2003). NODs: Intracellular Proteins Involved in Inflammation and Apoptosis. Nat. Rev. Immunol. 3, 371–382. 10.1038/nri1086 12766759

[B45] Jain N. Moeller J. Vogel V. (2019). Mechanobiology of Macrophages: How Physical Factors Coregulate Macrophage Plasticity and Phagocytosis. Annu. Rev. Biomed. Eng. 21, 267–297. 10.1146/annurev-bioeng-062117-121224 31167103

[B46] Jay T. R. Miller C. M. Cheng P. J. Graham L. C. Bemiller S. Broihier M. L. (2015). TREM2 Deficiency Eliminates TREM2+ Inflammatory Macrophages and Ameliorates Pathology in Alzheimer's Disease Mouse Models. J. Exp. Med. 212, 287–295. 10.1084/jem.20142322 25732305 PMC4354365

[B47] Jeong J.-W. Jin C.-Y. Kim G.-Y. Lee J.-D. Park C. Kim G.-D. (2010). Anti-inflammatory Effects of Cordycepin via Suppression of Inflammatory Mediators in BV2 Microglial Cells. Int. Immunopharmacology 10, 1580–1586. 10.1016/j.intimp.2010.09.011 20937401

[B48] Jetten N. Verbruggen S. Gijbels M. J. Post M. J. De Winther M. P. J. Donners M. M. P. C. (2014). Anti-inflammatory M2, but Not Pro-inflammatory M1 Macrophages Promote Angiogenesis *In Vivo* . Angiogenesis 17, 109–118. 10.1007/s10456-013-9381-6 24013945

[B49] Ji X.-C. Dang Y.-Y. Gao H.-Y. Wang Z.-T. Gao M. Yang Y. (2015). Local Injection of Lenti-BDNF at the Lesion Site Promotes M2 Macrophage Polarization and Inhibits Inflammatory Response after Spinal Cord Injury in Mice. Cell Mol Neurobiol 35, 881–890. 10.1007/s10571-015-0182-x 25840805 PMC11486196

[B50] Kang S. Nakanishi Y. Kioi Y. Okuzaki D. Kimura T. Takamatsu H. (2018). Semaphorin 6D Reverse Signaling Controls Macrophage Lipid Metabolism and Anti-inflammatory Polarization. Nat. Immunol. 19, 561–570. 10.1038/s41590-018-0108-0 29777213

[B51] Khandia R. K. Munjal A. M. N. Iqbal H. Dhama K. (2017). Heat Shock Proteins: Therapeutic Perspectives in Inflammatory Disorders. Recent Patents Inflamm. Allergy Drug Discov. 10, 94–104. 10.2174/1872213X10666161213163301 27978789

[B52] Kobayakawa K. Ohkawa Y. Yoshizaki S. Tamaru T. Saito T. Kijima K. (2019). Macrophage Centripetal Migration Drives Spontaneous Healing Process after Spinal Cord Injury. Sci. Adv. 5, eaav5086. 10.1126/sciadv.aav5086 31106270 PMC6520026

[B53] Kong F.-Q. Zhao S.-J. Sun P. Liu H. Jie J. Xu T. (2020). Macrophage MSR1 Promotes the Formation of Foamy Macrophage and Neuronal Apoptosis after Spinal Cord Injury. J. Neuroinflammation 17, 62. 10.1186/s12974-020-01735-2 32066456 PMC7027125

[B54] Kong X. Gao J. (2017). Macrophage Polarization: a Key Event in the Secondary Phase of Acute Spinal Cord Injury. J. Cel. Mol. Med. 21, 941–954. 10.1111/jcmm.13034 PMC5387136 27957787

[B55] Kroner A. Rosas Almanza J. (2019). Role of Microglia in Spinal Cord Injury. Neurosci. Lett. 709, 134370. 10.1016/j.neulet.2019.134370 31283964

[B56] Kuroda H. Jamiyan T. (2021). Tumor Microenvironment in Triple-Negative Breast Cancer: the Correlation of Tumor-Associated Macrophages and Tumor-Infiltrating Lymphocytes. Clin. Transl Oncol. 23, 2513–2525. 10.1007/s12094-021-02652-3 34089486 PMC8557183

[B57] Lech M. Anders H.-J. (2013). Macrophages and Fibrosis: How Resident and Infiltrating Mononuclear Phagocytes Orchestrate All Phases of Tissue Injury and Repair. Biochim. Biophys. Acta (Bba) - Mol. Basis Dis. 1832, 989–997. 10.1016/j.bbadis.2012.12.001 23246690

[B58] Li Q. Barres B. A. (2018). Microglia and Macrophages in Brain Homeostasis and Disease. Nat. Rev. Immunol. 18, 225–242. 10.1038/nri.2017.125 29151590

[B59] Lin S.-S. Tang Y. Illes P. Verkhratsky A. (2020). The Safeguarding Microglia: Central Role for P2Y12 Receptors. Front. Pharmacol. 11, 627760. 10.3389/fphar.2020.627760 33519493 PMC7840491

[B60] Liu J. Li K. Zhou J. Sun T. Yang C. Wei J. (2019). Bisperoxovanadium Induces M2-type Macrophages and Promotes Functional Recovery after Spinal Cord Injury. Mol. Immunol. 116, 56–62. 10.1016/j.molimm.2019.09.022 31605961

[B61] London A. Itskovich E. Benhar I. Kalchenko V. Mack M. Jung S. (2011). Neuroprotection and Progenitor Cell Renewal in the Injured Adult Murine Retina Requires Healing Monocyte-Derived Macrophages. J. Exp. Med. Jan 208, 23–39. 10.1084/jem.20101202 PMC3023128 21220455

[B62] Longbrake E. E. Lai W. Ankeny D. P. Popovich P. G. (2007). Characterization and Modeling of Monocyte-Derived Macrophages after Spinal Cord Injury. J. Neurochem. 102, 1083–1094. 10.1111/j.1471-4159.2007.04617.x 17663750

[B63] Lu H. Wu Y. Shao X. Zhou S. Jiang Y. Chen R. (2018). ANG II Facilitated CD11 + Ly6C Hi Cells Reprogramming into M1‐like Macrophage through Erk1/2 or p38‐Stat3 Pathway and Involved in EAM. J. Leukoc. Biol. 103, 719–730. 10.1002/JLB.3A0617-264RR 29350825

[B64] Ma S.-F. Chen Y.-J. Zhang J.-X. Shen L. Wang R. Zhou J.-S. (2015). Adoptive Transfer of M2 Macrophages Promotes Locomotor Recovery in Adult Rats after Spinal Cord Injury. Brain Behav. Immun. 45, 157–170. 10.1016/j.bbi.2014.11.007 25476600

[B65] Mabon P. J. Weaver L. C. Dekaban G. A. (2000). Inhibition of Monocyte/Macrophage Migration to a Spinal Cord Injury Site by an Antibody to the Integrin αD: A Potential New Anti-inflammatory Treatment. Exp. Neurol. 166, 52–64. 10.1006/exnr.2000.7488 11031083

[B66] Margeta M. A. Lad E. M. Proia A. D. (2018). CD163+ Macrophages Infiltrate Axon Bundles of Postmortem Optic Nerves with Glaucoma. Graefes Arch. Clin. Exp. Ophthalmol. 256, 2449–2456. 10.1007/s00417-018-4081-y 30073622 PMC6221945

[B67] Martin P. D'Souza D. Martin J. Grose R. Cooper L. Maki R. (2003). Wound Healing in the PU.1 Null Mouse-Tissue Repair Is Not Dependent on Inflammatory Cells. Curr. Biol. 13, 1122–1128. 10.1016/s0960-9822(03)00396-8 12842011

[B68] Maurer M. von Stebut E. (2004). Macrophage Inflammatory Protein-1. Int. J. Biochem. Cel Biol. 36, 1882–1886. 10.1016/j.biocel.2003.10.019 15203102

[B69] McKerracher L. Rosen K. M. (2015). MAG, Myelin and Overcoming Growth Inhibition in the CNS. Front. Mol. Neurosci. 8, 51. 10.3389/fnmol.2015.00051 26441514 PMC4561339

[B70] Mebius R. E. Kraal G. (2005). Structure and Function of the Spleen. Nat. Rev. Immunol. 5, 606–616. 10.1038/nri1669 16056254

[B71] Milich L. M. Ryan C. B. Lee J. K. (2019). The Origin, Fate, and Contribution of Macrophages to Spinal Cord Injury Pathology. Acta Neuropathol. 137, 785–797. 10.1007/s00401-019-01992-3 30929040 PMC6510275

[B72] Mills C. (2012). M1 and M2 Macrophages: Oracles of Health and Disease. Crit. Rev. Immunol. 32, 463–488. 10.1615/critrevimmunol.v32.i6.10 23428224

[B73] Miron V. E. Boyd A. Zhao J.-W. Yuen T. J. Ruckh J. M. Shadrach J. L. (2013). M2 Microglia and Macrophages Drive Oligodendrocyte Differentiation during CNS Remyelination. Nat. Neurosci. 16, 1211–1218. 10.1038/nn.3469 23872599 PMC3977045

[B74] Morganti-Kossmann M. C. Satgunaseelan L. Bye N. Kossmann T. (2007). Modulation of Immune Response by Head Injury. Injury 38, 1392–1400. 10.1016/j.injury.2007.10.005 18048036

[B75] Motwani M. P. Gilroy D. W. (2015). Macrophage Development and Polarization in Chronic Inflammation. Semin. Immunol. 27, 257–266. 10.1016/j.smim.2015.07.002 26216597

[B76] Naeini M. B. Momtazi-Borojeni A. A. Ganjali S. Kontush A. Jaafari M. R. Sahebkar A. (2021). Phosphatidylserine-containing Liposomes: Therapeutic Potentials against Hypercholesterolemia and Atherosclerosis. Eur. J. Pharmacol. 908, 174308. 10.1016/j.ejphar.2021.174308 34245747

[B77] Nakai K. (2021). Multiple Roles of Macrophage in Skin. J. Dermatol. Sci. 104 (1), 2–10. 10.1016/j.jdermsci.2021.08.008 34493430

[B78] Nakajima H. Honjoh K. Watanabe S. Kubota A. Matsumine A. (2020). Distribution and Polarization of Microglia and Macrophages at Injured Sites and the Lumbar Enlargement after Spinal Cord Injury. Neurosci. Lett. 737, 135152. 10.1016/j.neulet.2020.135152 32531528

[B79] Niemeyer M. J. S. Lokerman R. D. Sadiqi S. van Heijl M. Houwert R. M. van Wessem K. J. P. (2020). Epidemiology of Traumatic Spinal Cord Injury in the Netherlands: Emergency Medical Service, Hospital, and Functional Outcomes. Top. Spinal Cord Inj. Rehabil. 26, 243–252. 10.46292/sci20-00002 33536729 PMC7831280

[B80] Novak M. L. Koh T. J. (2013). Phenotypic Transitions of Macrophages Orchestrate Tissue Repair. Am. J. Pathol. 183, 1352–1363. 10.1016/j.ajpath.2013.06.034 24091222 PMC3969506

[B81] Oyinbo C. A. (2011). Secondary Injury Mechanisms in Traumatic Spinal Cord Injury: a Nugget of This Multiply cascade. Acta Neurobiol. Exp. (Wars) 71, 281–299. Available from: https://www.ncbi.nlm.nih.gov/pubmed/21731081 . 21731081 10.55782/ane-2011-1848

[B82] Parvin S. Williams C. R. Jarrett S. A. Garraway S. M. (2021). Spinal Cord Injury Increases Pro-inflammatory Cytokine Expression in Kidney at Acute and Sub-chronic Stages. Inflammation. 10.1007/s10753-021-01507-x PMC8616867 34417952

[B83] Perdiguero E. Sousa-Victor P. Ruiz-Bonilla V. Jardí M. Caelles C. Serrano A. L. (2011). p38/MKP-1-regulated AKT Coordinates Macrophage Transitions and Resolution of Inflammation during Tissue Repair. J. Cel Biol 195, 307–322. 10.1083/jcb.201104053 PMC3198158 21987635

[B84] Pickett G. E. Campos-Benitez M. Keller J. L. Duggal N. (2006). Epidemiology of Traumatic Spinal Cord Injury in Canada. Spine 31, 799–805. 10.1097/01.brs.0000207258.80129.03 16582854

[B85] Picoli C. C. Coimbra-Campos L. M. C. Guerra D. A. P. Silva W. N. Prazeres P. H. D. M. Costa A. C. (2019). Pericytes Act as Key Players in Spinal Cord Injury. Am. J. Pathol. 189, 1327–1337. 10.1016/j.ajpath.2019.03.008 31014955 PMC6717911

[B86] Popovich P. G. Guan Z. Wei P. Huitinga I. van Rooijen N. Stokes B. T. (1999). Depletion of Hematogenous Macrophages Promotes Partial Hindlimb Recovery and Neuroanatomical Repair after Experimental Spinal Cord Injury. Exp. Neurol. 158, 351–365. 10.1006/exnr.1999.7118 10415142

[B87] Prinz M. Masuda T. Wheeler M. A. Quintana F. J. (2021). Microglia and Central Nervous System-Associated Macrophages-From Origin to Disease Modulation. Annu. Rev. Immunol. 39, 251–277. 10.1146/annurev-immunol-093019-110159 33556248 PMC8085109

[B88] Przekora A. Juszkiewicz L. (2020). The Effect of Autologous Adipose Tissue-Derived Mesenchymal Stem Cells' Therapy in the Treatment of Chronic Posttraumatic Spinal Cord Injury in a Domestic Ferret Patient. Cel Transpl. 29, 096368972092898. 10.1177/0963689720928982 PMC7563821 32441545

[B89] Ren Y. Young W. (2013). Managing inflammation after spinal cord injury through Manipulation of Macrophage Function. Neural Plasticity 2013, 1–9. 10.1155/2013/945034 PMC3833318 24288627

[B90] Sahni V. Mukhopadhyay A. Tysseling V. Hebert A. Birch D. McGuire T. L. (2010). BMPR1a and BMPR1b Signaling Exert Opposing Effects on Gliosis after Spinal Cord Injury. J. Neurosci. 30, 1839–1855. 10.1523/JNEUROSCI.4459-09.2010 20130193 PMC3093918

[B91] Samantaray S. Das A. Matzelle D. C. Yu S. P. Wei L. Varma A. (2016). Administration of Low Dose Estrogen Attenuates Gliosis and Protects Neurons in Acute Spinal Cord Injury in Rats. J. Neurochem. 136, 1064–1073. 10.1111/jnc.13464 26662641 PMC5374720

[B92] Selvarajah S. Hammond E. R. Haider A. H. Abularrage C. J. Becker D. Dhiman N. (2014). The burden of Acute Traumatic Spinal Cord Injury Among Adults in the united states: an Update. J. Neurotrauma 31, 228–238. 10.1089/neu.2013.3098 24138672

[B93] Shang Q. Chen J. Hu Y. Yang X. Hu L. Liu C. (2021). Facile Fabrication of Superhydrophobic Cross-Linked Nanocellulose Aerogels for Oil-Water Separation. Polymers 13, 625. 10.3390/polym13040625 33669607 PMC7921982

[B94] Shechter R. London A. Varol C. Raposo C. Cusimano M. Yovel G. (2009). Infiltrating Blood-Derived Macrophages Are Vital Cells Playing an Anti-inflammatory Role in Recovery from Spinal Cord Injury in Mice. Plos Med. 6, e1000113. 10.1371/journal.pmed.1000113 19636355 PMC2707628

[B95] Shechter R. Raposo C. London A. Sagi I. Schwartz M. (2011). The Glial Scar-Monocyte Interplay: a Pivotal Resolution Phase in Spinal Cord Repair. PLoS One 6, e27969. 10.1371/journal.pone.0027969 22205935 PMC3244386

[B96] Shimobayashi M. Albert V. Woelnerhanssen B. Frei I. C. Weissenberger D. Meyer-Gerspach A. C. (2018). Insulin Resistance Causes Inflammation in Adipose Tissue. J. Clin. Invest. 128, 1538–1550. 10.1172/JCI96139 29528335 PMC5873875

[B97] Sica A. Erreni M. Allavena P. Porta C. (2015). Macrophage Polarization in Pathology. Cell. Mol. Life Sci. 72, 4111–4126. 10.1007/s00018-015-1995-y 26210152 PMC11113543

[B98] Song G. Yang R. Zhang Q. Chen L. Huang D. Zeng J. (2019). TGF-β Secretion by M2 Macrophages Induces Glial Scar Formation by Activating Astrocytes *In Vitro* . J. Mol. Neurosci. 69, 324–332. 10.1007/s12031-019-01361-5 31327154

[B99] Song J. Frieler R. A. Whitesall S. E. Chung Y. Vigil T. M. Muir L. A. (2021). Myeloid Interleukin-4 Receptor α Is Essential in Postmyocardial Infarction Healing by Regulating Inflammation and Fibrotic Remodeling. Am. J. Physiology-Heart Circulatory Physiol. 320, H323–H337. 10.1152/ajpheart.00251.2020 PMC7847075 33164548

[B100] Sun J. Zhang J. Li K. Zheng Q. Song J. Liang Z. (2020). Photobiomodulation Therapy Inhibit the Activation and Secretory of Astrocytes by Altering Macrophage Polarization. Cel Mol Neurobiol 40, 141–152. 10.1007/s10571-019-00728-x PMC11448964 31446561

[B101] Swirski F. K. Hilgendorf I. Robbins C. S. (2014). From Proliferation to Proliferation: Monocyte Lineage Comes Full circle. Semin. Immunopathol 36, 137–148. 10.1007/s00281-013-0409-1 24435095 PMC3991755

[B102] Swirski F. K. Nahrendorf M. Etzrodt M. Wildgruber M. Cortez-Retamozo V. Panizzi P. (2009). Identification of Splenic Reservoir Monocytes and Their Deployment to Inflammatory Sites. Science 325, 612–616. 10.1126/science.1175202 19644120 PMC2803111

[B103] Tom V. J. Steinmetz M. P. Miller J. H. Doller C. M. Silver J. (2004). Studies on the Development and Behavior of the Dystrophic Growth Cone, the Hallmark of Regeneration Failure, in an *In Vitro* Model of the Glial Scar and after Spinal Cord Injury. J. Neurosci. 24, 6531–6539. 10.1523/JNEUROSCI.0994-04.2004 15269264 PMC6729861

[B104] Urbanski K. Ludew D. Filip G. Filip M. Sagan A. Szczepaniak P. (2017). CD14+CD16++ "nonclassical" Monocytes Are Associated with Endothelial Dysfunction in Patients with Coronary Artery Disease. Thromb. Haemost. 117, 971–980. 10.1160/TH16-08-0614 28229168

[B105] Vismara I. Papa S. Veneruso V. Mauri E. Mariani A. De Paola M. (2020). Selective Modulation of A1 Astrocytes by Drug-Loaded Nano-Structured Gel in Spinal Cord Injury. ACS Nano 14, 360–371. 10.1021/acsnano.9b05579 31887011

[B106] Wang J. L. Luo X. Liu L. (2019). Targeting CARD6 Attenuates Spinal Cord Injury (SCI) in Mice through Inhibiting Apoptosis, Inflammation and Oxidative Stress Associated ROS Production. Aging 11, 12213–12235. 10.18632/aging.102561 31841440 PMC6949089

[B107] Wang X. Cao K. Sun X. Chen Y. Duan Z. Sun L. (2015). Macrophages in Spinal Cord Injury: Phenotypic and Functional Change from Exposure to Myelin Debris. Glia 63, 635–651. 10.1002/glia.22774 25452166 PMC4331228

[B108] Wanner I. B. Anderson M. A. Song B. Levine J. Fernandez A. Gray-Thompson Z. (2013). Glial Scar Borders Are Formed by Newly Proliferated, Elongated Astrocytes that Interact to Corral Inflammatory and Fibrotic Cells via STAT3-dependent Mechanisms after Spinal Cord Injury. J. Neurosci. 33, 12870–12886. 10.1523/JNEUROSCI.2121-13.2013 23904622 PMC3728693

[B109] Watanabe S. Alexander M. Misharin A. V. Budinger G. R. S. (2019). The Role of Macrophages in the Resolution of Inflammation. J. Clin. Invest. 129, 2619–2628. 10.1172/JCI124615 31107246 PMC6597225

[B110] Wattananit S. Tornero D. Graubardt N. Memanishvili T. Monni E. Tatarishvili J. (2016). Monocyte-Derived Macrophages Contribute to Spontaneous Long-Term Functional Recovery after Stroke in Mice. J. Neurosci. 36, 4182–4195. 10.1523/JNEUROSCI.4317-15.2016 27076418 PMC6601783

[B111] Wilson J. R. Cronin S. Fehlings M. G. Kwon B. K. Badhiwala J. H. Ginsberg H. J. (2020). Epidemiology and Impact of Spinal Cord Injury in the Elderly: Results of a Fifteen-Year Population-Based Cohort Study. J. Neurotrauma 37, 1740–1751. 10.1089/neu.2020.6985 32292120

[B112] Wu D. Klaw M. C. Connors T. Kholodilov N. Burke R. E. Tom V. J. (2015). Expressing Constitutively Active Rheb in Adult Neurons after a Complete Spinal Cord Injury Enhances Axonal Regeneration beyond a Chondroitinase-Treated Glial Scar. J. Neurosci. 35, 11068–11080. 10.1523/JNEUROSCI.0719-15.2015 26245968 PMC4524976

[B113] Xia H.-f. Zhu J.-y. Wang J.-n. Ren J.-g. Cai Y. Wang F.-q. (2017). Association of ATF4 Expression with Tissue Hypoxia and M2 Macrophage Infiltration in Infantile Hemangioma. J. Histochem. Cytochem. 65, 285–294. 10.1369/0022155417694872 28438094 PMC5407535

[B114] Xu B. Park D. Ohtake Y. Li H. Hayat U. Liu J. (2015). Role of CSPG Receptor LAR Phosphatase in Restricting Axon Regeneration after CNS Injury. Neurobiol. Dis. 73, 36–48. 10.1016/j.nbd.2014.08.030 25220840 PMC4427014

[B115] Yang L. Ge D. Chen X. Jiang C. Zheng S. (2018). miRNA-544a Regulates the Inflammation of Spinal Cord Injury by Inhibiting the Expression of NEUROD4. Cell Physiol Biochem 51, 1921–1931. 10.1159/000495717 30513512

[B116] Yao A. Liu F. Chen K. Tang L. Liu L. Zhang K. (2014). Programmed Death 1 Deficiency Induces the Polarization of Macrophages/microglia to the M1 Phenotype after Spinal Cord Injury in Mice. Neurotherapeutics 11, 636–650. 10.1007/s13311-013-0254-x 24853068 PMC4121443

[B117] Zhang J. Li Y. Duan Z. Kang J. Chen K. Li G. (2020). The Effects of the M2a Macrophage-Induced Axonal Regeneration of Neurons by Arginase 1. Biosci. Rep. 40, 40. 10.1042/BSR20193031 PMC7012653 31994698

[B118] Zhang Q. Zhu C. Li X. Shi Y. Zhang Z. (2021). CCR2 Downregulation Attenuates Spinal Cord Injury by Suppressing Inflammatory Monocytes. Synapse 75, e22191. 10.1002/syn.22191 33098174

[B119] Zhao Y. Zou W. Du J. Zhao Y. (2018). The Origins and Homeostasis of Monocytes and Tissue‐resident Macrophages in Physiological Situation. J. Cel Physiol 233, 6425–6439. 10.1002/jcp.26461 29323706

[B120] Zheng Q. Zhang J. Zuo X. Sun J. Liang Z. Hu X. (2021). Photobiomodulation Promotes Neuronal Axon Regeneration after Oxidative Stress and Induces a Change in Polarization from M1 to M2 in Macrophages via Stimulation of CCL2 in Neurons: Relevance to Spinal Cord Injury. J. Mol. Neurosci. 71, 1290–1300. 10.1007/s12031-020-01756-9 33417168

[B121] Zhou J. Li Z. Wu T. Zhao Q. Zhao Q. Cao Y. (2020). LncGBP9/miR-34a axis Drives Macrophages toward a Phenotype Conducive for Spinal Cord Injury Repair via STAT1/STAT6 and SOCS3. J. Neuroinflammation 17, 134. 10.1186/s12974-020-01805-5 32345320 PMC7187522

[B122] Zhou Q. Xiang H. Li A. Lin W. Huang Z. Guo J. (2019). Activating Adiponectin Signaling with Exogenous AdipoRon Reduces Myelin Lipid Accumulation and Suppresses Macrophage Recruitment after Spinal Cord Injury. J. Neurotrauma 36, 903–918. 10.1089/neu.2018.5783 30221582

[B123] Zhou Y. Do D. C. Ishmael F. T. Squadrito M. L. Tang H. M. Tang H. L. (2018). Mannose Receptor Modulates Macrophage Polarization and Allergic Inflammation through miR-511-3p. J. Allergy Clin. Immunol. 141, 350–364. 10.1016/j.jaci.2017.04.049 28629744 PMC5944850

[B124] Zhu Y. Soderblom C. Krishnan V. Ashbaugh J. Bethea J. R. Lee J. K. (2015). Hematogenous Macrophage Depletion Reduces the Fibrotic Scar and Increases Axonal Growth after Spinal Cord Injury. Neurobiol. Dis. 74, 114–125. 10.1016/j.nbd.2014.10.024 25461258 PMC4323620

[B125] Zrzavy T. Schwaiger C. Wimmer I. Berger T. Bauer J. Butovsky O. (2021). Acute and Non-resolving Inflammation Associate with Oxidative Injury after Human Spinal Cord Injury. Brain. 144, 144–161. 10.1093/brain/awaa360 33578421 PMC7880675

